# Surface flattening of the human left atrium and proof-of-concept clinical applications^☆^

**DOI:** 10.1016/j.compmedimag.2014.01.004

**Published:** 2014-06

**Authors:** Rashed Karim, YingLiang Ma, Munjung Jang, R. James Housden, Steven E. Williams, Zhong Chen, Asghar Ataollahi, Kaspar Althoefer, C. Aldo Rinaldi, Reza Razavi, Mark D. O’Neill, Tobias Schaeftter, Kawal S. Rhode

**Affiliations:** a Division of Imaging Sciences and Biomedical Engineering, King's College London, SE1 7EH, UK; b Department of Cardiology, Guy's and St. Thomas’ NHS Foundation Trust, London SE1 7EH, UK; c Centre for Robotics Research, Department of Informatics, King's College London, SE1 7EH, UK

**Keywords:** Left atrial surface parameterization, Lesion visualization, Surface flattening, Catheter ablation planning and navigation, Atrial fibrillation

## Abstract

Surface flattening in medical imaging has seen widespread use in neurology and more recently in cardiology to describe the left ventricle using the bull's-eye plot. The method is particularly useful to standardize the display of functional information derived from medical imaging and catheter-based measurements. We hypothesized that a similar approach could be possible for the more complex shape of the left atrium (LA) and that the surface flattening could be useful for the management of patients with atrial fibrillation (AF). We implemented an existing surface mesh parameterization approach to flatten and unfold 3D LA models. Mapping errors going from 2D to 3D and the inverse were investigated both qualitatively and quantitatively using synthetic data of regular shapes and computer tomography scans of an anthropomorphic phantom. Testing of the approach was carried out using data from 14 patients undergoing ablation treatment for AF. 3D LA meshes were obtained from magnetic resonance imaging and electroanatomical mapping systems. These were unfolded using the developed approach and used to demonstrate proof-of-concept applications, such as the display of scar information, electrical information and catheter position. The work carried out shows that the unfolding of complex cardiac structures, such as the LA, is feasible and has several potential clinical uses for the management of patients with AF.

## 1 Introduction

### 1.1 Background

Planar mapping or surface parameterization of three-dimensional (3D) meshes is a well studied problem in computer graphics [1]. It has a wide range of applications in various fields of science and engineering with the most important application being mapping textures to polygonal mesh models for enhancing their visual quality. Parameterizations are known to introduce distortions in either angles or areas and it is desirable that a mapping minimizes these distortions. An *isometric* mapping is one that preserves distances and is almost impossible to achieve for complex surfaces. Others such as *conformal* (angle-preserving) or *authalic* (area-preserving) are possible. Floater [2–4] produced several works in surface parameterization by extending a previously known geometrical theorem due to Tutte [5]. Floater's methods imposed fixed boundaries in the resulting parameterization. Free boundary parameterization methods have been proposed by Scheffer and de Sturler [6] and later by Levy et al. [7]. Desbrun et al. [8] employed conformal energy functions with linear solutions capable of both free and fixed boundary parameterization.

Surface parameterization has been applied to problems arising in medical image processing. Planar mapping of the cerebral cortex has important applications and have been proposed in Hurdal et al. [9], Haker et al. [10] and Gu et al. [11] for computing conformal maps. The use of Ricci flow in flattening the cortex has also been investigated [12]. More recently, planar maps have also been commonly used for registering images of the brain, e.g. [13]. A common technique is to map the cortex to a spherical surface. The sphere provides a standard platform for comparison before registration can be accomplished. This is the method implemented in FreeSurfer [14], a leading and widely used tool for brain surface reconstruction. However, FreeSurfer suffers from a severe computational burden causing it to be very slow. Most of the existing methods rely on some form of dimensionality reduction and this usually involves solving an eigen value problem. The size of the mesh can often be a limiting factor and there is usually a trade-off between speed and mesh size. Often this means decimating a mesh and thus lowering the resolution of the underlying data.

This manuscript concerns application of planar mapping to the heart. The use of this has been so far restricted to the relatively simply shaped left ventricle (LV). The LV myocardial wall is often transformed into a planar *bull's-eye* plot [15]. This is a polar plot of different sections of the wall that allows simplification and standardization. It can be particularly useful for visualization and reporting of functional information about the LV, such as wall motion, perfusion and distribution of scarring. The bull's-eye plot has been adopted by the American Heart Association as the standard way to represent the LV. In this manuscript we implement, validate and test planar mapping of the more complex left atrium (LA) to assist in the management of patients with atrial fibrillation (AF).

### 1.2 Clinical motivation

AF is the most common heart rhythm disturbance and originates in the LA. It affects approximately 2.3 million people in the USA [16]. Since it was shown that ectopic beats from within the pulmonary veins (PV) commonly initiate AF [17], catheter ablation strategies have evolved to electrically isolate the PVs from the LA body. By controlled destruction of cardiac tissue in a circumferential pattern around the left and right PV pairs, the PVs are electrically disconnected from the LA and therefore cannot influence atrial electrical behaviour. Although this treatment can provide a cure for many patients, arrhythmia recurrences are common, particularly in patients with persistent AF [18–20], leading to a repeat procedure in up to 50% of this challenging patient group to achieve the best clinical outcomes.

Recurrences of AF following a circumferential PV isolation procedure are invariably associated with recovery of PV to LA conduction which is dependent largely on a failure to achieve continuous and permanent lesions. Multiple factors influence the operator's ability to achieve such a lesion including, but not limited to, catheter stability, power, tissue thickness, contact and temperature achieved, force and duration of radiofrequency (RF) application, cardiac motion and local heat sink effect. Many of the factors impeding effective lesion delivery can be mitigated in part by operator experience but they certainly cannot be eliminated [21] as evidenced by high volume centres continuing to report levels of arrhythmia recurrence in excess of 10–15% at five years after ablation [19,20]. Clearly, there remains a need to identify ways in which improvements in ablation technique can lead to better clinical outcomes.

Catheter ablation procedures for AF are performed by cardiac electrophysiologists. The vast majority of cardiac electrophysiologists utilize an electroanatomical mapping system to guide the procedure [21]. Both the CARTO (Biosense Webster) and Velocity (St. Jude Medical) systems permit reconstruction of an anatomical representation of the LA, electrophysiological characterization of the chamber (voltage, activation time) and display in real-time of the catheter position within the chamber. 3D electroanatomical models of the target cardiac chambers obtained by live catheter tracking, pre-procedural imaging, such as magnetic resonance imaging (MRI) or computed tomography (CT), or a combination of both are used to guide the interventions. In these systems, 3D models displayed on a two-dimensional (2D) screen require frequent interaction from the end-user for displaying the current region of interest. We hypothesized that the use of planar mapping of the LA could assist in the display of the anatomical and functional information that is commonly used in the management of patients with AF.

### 1.3 Proposed work

In earlier work [22] that focused on planar mapping of the LV, we briefly demonstrated that a B-spline based LA flattening may be possible. In this work we investigate a surface flattening technique developed primarily for texture mapping in computer graphics [8] and apply it to flatten 3D LA meshes. The mathematical background is described in Section 2.1. We qualitatively and quantitatively validated the technique by application to synthetic data of regular shapes (Section 2.2.1) and to an anthropomorphic phantom imaged with CT (Section 2.2.2). Finally we test our method using 14 data sets from patients that underwent RF ablation for treatment of atrial fibrillation and use these data to demonstrate several proof-of-concept applications of the planar mapping (Section 2.2.3).

## 2 Methods

### 2.1 Mathematical framework for surface unfolding

We define here the problem of unfolding and flattening the LA mesh. Essentially this is a parameterization of a piece-wise mesh. Given a piecewise linear mesh *M* of the LA, the problem of mesh parameterization is to obtain a linear mapping *φ* between M and a planar triangulation $U\in {\mathbb{R}}^{2}$. The mapping is isomorphic meaning *U* must inherit the same intrinsic and structural properties as *M*. Mesh *M* is non-closed due to a cut made at the mitral valve annulus and contains holes at each ostium since the PVs must be truncated before the unfold. The 3D position of the *i*th vertex in the mesh is denoted by ${\text{x}}_{i}={\left(x_{i},y_{i},z_{i}\right)}^{t}$ and in the 2D mesh *U* as ${\text{u}}_{i}={\left(u_{i},v_{i}\right)}^{t}$. The mapping *φ* creates a one-to-one correspondence between the 3D mesh and its 2D unfold, essentially flattening the mesh to a planar map.

Since the LA is an anatomical structure with some distinct anatomical features, such as the appendage, it is important to preserve, when unfolding, as much of the natural or *intrinsic* properties of the LA mesh as possible. The distortion measure *E* between *M* and *U* is a functional that takes in as input two triangulations and returns a real value:

(1) E:T×T→ℝ

**Fig. 1:**
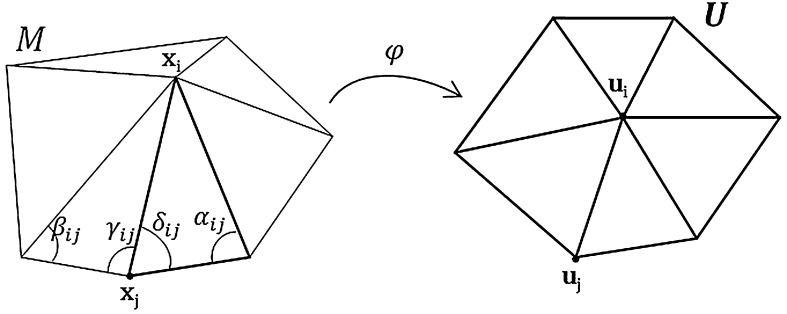
LA mesh patch or 1-ring neighbourhoods **M** in 3D with its associated flattened planar 1-ring U in 2D.

This distortion measure evaluates how much the structural properties of the meshes are distorted due to the unfolding. In [8], a distortion measure was developed by ensuring a few basic properties are fulfilled by a predefined functional. The two most important properties relevant to LA meshes are listed here: (1) invariance due to rigid transformations of the mesh, and (2) preserves continuity due to finer triangulations. The former is important as two meshes exactly alike but having undergone rotations and translations must yield a zero distortion, thereby allowing the parameterization to be independent of any rigid transformations. The latter ensures that this discrete version of distortion (due to the discrete nature of LA meshes) converges to its continuous form as we get finer and finer triangulations of the mesh. We restrict our discussion of distortion measures to *1-ring neighbourhoods*. A 1-ring is a subset of the 3D LA mesh, consisting of a vertex and all its adjacent triangles. See Fig. 1 for an illustration of a 3D 1-ring neighbourhood with its corresponding unfold planar 2D 1-ring. The distortion measure and thus the parameterization, discussed further in this text, is one which preserves angles and areas of 1-ring neighbourhoods.

#### 2.1.1 Angle-preservation

An angle-preserving (i.e. conformal) distortion measure can be developed by quantifying shape distortions of the mapping function. In Differential Geometry, due to the *minimal surface* problem, it is well known that the minimization of a so-called Dirichlet energy leads to a conformal mapping [23]. The Dirichlet energy can be defined on a mesh patch and captures angle distortions between corresponding triangles of the patch in 3D and 2D. It can be shown to fulfil the desirable properties outlined above for the unfold [23]. The total Dirichlet energy for the patch is given by:

(2) $$E_{A}=\sum_{\text{ring}\;\text{edges}(i,j)}(\cot\;\alpha_{ij}+\cot\;\beta_{ij})|{\text{u}}_{i}-{\text{u}}_{j}{|}^{2}$$

where the length $\left|{\text{u}}_{i}-{\text{u}}_{j}\right|$ is in the 2D parameter domain and angle $\alpha_{ij}$ is the opposite angle to $\left({\text{x}}_{i},{\text{x}}_{j}\right)$ in 3D (see Fig. 1). The derivation is omitted here but is a straightforward differentiation of a linear map and can be found in [23]. There are two advantages of using this function: (1) it depends only on the angle of the 3D mesh (i.e. cotangent term), and (2) the first-order derivative of the energy term is conveniently linear:

(3) $$\frac{\partial E_{A}}{\partial {\text{u}}_{i}}=\sum_{\text{ring}\;\text{edges}(i,j)}(\cot\;\alpha_{ij}+\cot\;\beta_{ij})({\text{u}}_{i}-{\text{u}}_{j})=0$$

The optimal placement of vertex **u**_*i*_ can thus be solved by solving the linear system in Eq. (3). The Dirichlet energy in Eq. (2) thus gives a measure of distortion in-terms of the angles between the mesh patch in 3D and its unfold in 2D. If the Dirichlet energy is minimized, a conformal (i.e. angle preserving) transformation is possible, however, since we aim to fix the border of the 2D unfold to a square border, achieving a fully conformal transformation may be impossible. This Dirichlet energy term becomes part of the final distortion measure.

#### 2.1.2 Area-preservation

**Fig. 2:**
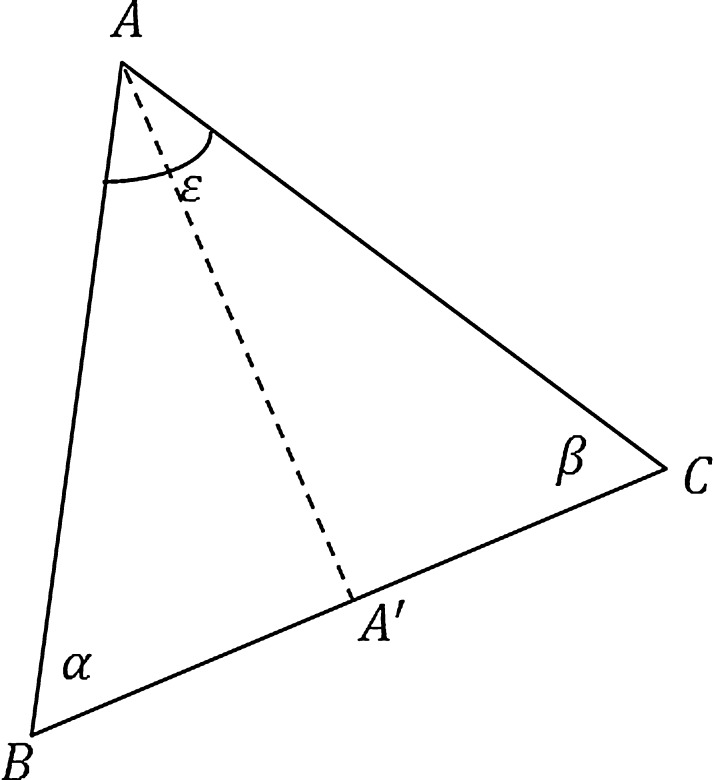
Deriving the gradient of the tip angle of a 3D mesh patch.

Aside from preserving angles, there is also an area-preserving component. Consider the tip angle around **x**_*i*_ of the 3D mesh *M* in Fig. 1; the gradient of this angle can be easily derived from its individual triangles shown in Fig. 2, the gradient of the angle *ɛ* w.r.t. placement of vertex *A* is given by:

(4) $$\nabla \varepsilon =\frac{\cot\;\alpha }{|AB{|}^{2}}\text{AB}+\frac{\cot\;\beta }{|AC{|}^{2}}\text{AC}$$

Note that both terms of ▿*ɛ* in Eq. (4) are functions of the triangle's area. For instance if projection of *C* onto *AB* is *C*′ then the first term's co-efficient $\cot\;\alpha /|AB{|}^{2}$ is equal to the area of $\Delta CBC^{\prime }/2T^{2}$ where *T* is the area of Δ*ABC*. By summing angles around the 3D mesh in *M*, it can be shown that:

(5) $$\nabla \theta =\sum_{j\in N(i)}\frac{\cot\;\gamma_{ij}+\cot\;\delta_{ij}}{|{\text{x}}_{i}-{\text{x}}_{j}{|}^{2}}({\text{x}}_{j}-{\text{x}}_{i})$$

where ▿ is gradient w.r.t. vertex **x**_*i*_ and *θ* is the total angle around **x**_*i*_ and not necessarily 2*π* due to its positioning in 3D. Note that similar to Eq. (4), the co-efficients in Eq. (5) are functions of the area.

Following from Eq. (5) Desbrun et al. [8] obtain a novel distortion measure:

(6) $$E_{x}=\sum_{j\in N(i)}\frac{\cot\;\gamma_{ij}+\cot\;\delta_{ij}}{|{\text{x}}_{i}-{\text{x}}_{j}{|}^{2}}{\left({\text{u}}_{i}-{\text{u}}_{j}\right)}^{2}$$

where angles *γ*_*ij*_ and *δ*_*ij*_ are defined in Fig. 1. This combines area of the mesh *M* and its unfolding *U*. Note the only unknown is vertex **u**_*i*_ and thus its placement in the parameter space. Once again, the optimal placement of **u**_*i*_ can be determined by solving $\partial E_{X}/\partial {\text{u}}_{i}=0$ and this conveniently results in a linear problem.

#### 2.1.3 Final parameterization

The two distortion measures described above are combined into a single measure. In addition to this a fixed boundary condition is imposed requiring the border of the unfolding to be a square. The combined distortion measure can thus be written as:

(7) $$E=\lambda E_{A}+(1-\lambda )E_{X}$$

where *λ* is a constant weighing the influence of each distortion measure. To obtain the final parameterization, positions of internal vertices **u**_***i***_ in the 2D unfold are solved for. The boundary conditions are stated beforehand, i.e. the square border of the parameter space is matched with the border of the 3D LA mesh. The steps to cut open the LA mesh and thus define its borders are described below. The optimal positions of all **u**_***i***_ can be obtained by minimizing the combined distortion measure and thus solving a sparse linear system following from $\partial E_{X}/\partial {\text{u}}_{i}=0$ and $\partial E_{A}/\partial {\text{u}}_{i}=0$:

(8) MU=C

where **M** is the co-efficient matrix carrying co-efficients of the respective distortion measures, i.e. $\partial E_{A}/\partial {\text{u}}_{i}$ and $\partial E_{X}/\partial {\text{u}}_{i}$. **C** is the vector of boundary conditions: ${\left[0\;{\text{C}}^{\text{boundary}}\right]}^{T}$ and the positions of internal vertices are solved for in **U**. The system can be efficiently solved using Conjugate Gradient and SSOR (Symmetric Successive Over-relaxation) preconditioning [24]. For this work, the implementation of the surface parameterization of [8] is the one found in the Computational Geometry Algorithms Library (CGAL) [25] with wrappers written in C++ and using VTK for visualization and mapping procedures.

#### 2.1.4 Method initialization

**Fig. 3:**
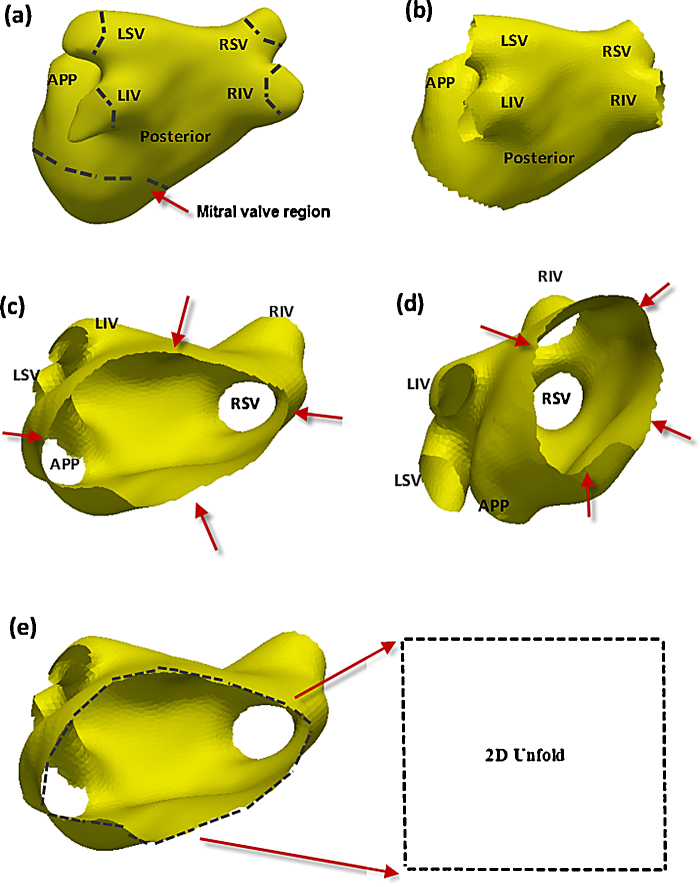
(a) Initialization of the unfolding process begins with a manual cut around the mitral valve annulus of the LA and four veins and appendage as indicated by dashed lines, (b) LA after the cut, (c) the border of the mitral valve cut as indicated using red arrows is matched with the 2D unfold's boundary. (d) Same as (c) but seen from a different view plane. (e) Boundary of mitral valve cut in 3D is mapped to boundary of unfold in 2D. This completes the initialization process. *Abbreviations*: RIV, right inferior vein; RSV, right superior vein; LIV, left inferior vein; LSV, left superior vein; APP, appendage.

Prior to computing the parameterization for the unfolding, boundary conditions must be set. The boundary of the 2D unfold must be mapped initially to a boundary in the 3D LA mesh. An ideal boundary is the LA mitral valve annulus where the LA joins the left ventricle. The LA is thus manually cut in this region and an illustration is provided in Fig. 3(a) showing the location of such a cut. The boundary of the cut is generally disc shaped owing to the LA's spherically shaped chamber (see Fig. 3(b)–(d)). The disc is mapped to the 2D unfold boundary (Fig. 3(e)) and this initializes the unfolding process. Furthermore, additional cuts are made at each PV and the appendage. However, this is only to map vein and appendage locations in the unfold with holes. The mitral valve cut affects the final parameterization result and thus the quality of unfold. As ablation lesions are only made in and around PVs, the cut is made appropriately to cover vital regions of the LA. The cut was performed using mesh editing software (MeshLab, ISTI – CNR, Pisa, Italy).

### 2.2 Experimental protocol

The proposed unfold technique was initially validated using synthetic regular shape data. It was then validated using data from an anthropomorphic glass heart phantom. Finally, it was applied to patient datasets.

#### 2.2.1 Regular shapes

**Fig. 4:**
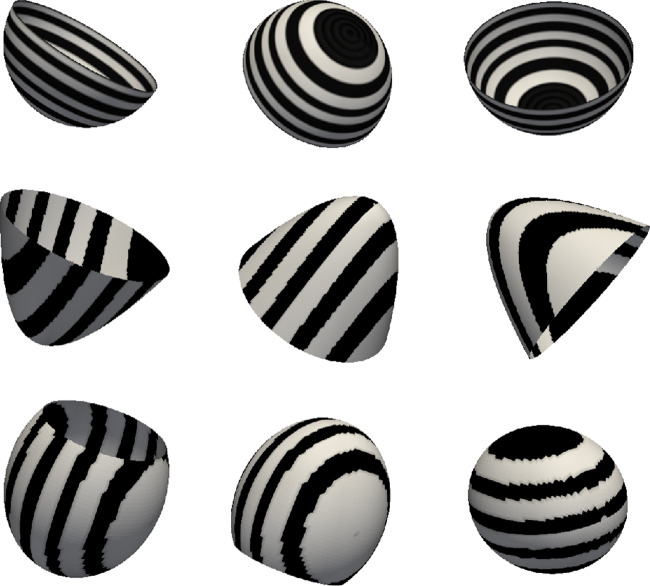
Common geometrical shapes with regular texture patterns that were used for evaluating the unfold technique. The shapes were a hemisphere (top row), paraboloid (middle row) and a truncated ellipsoid (bottom row).

To evaluate the extent of deformations with the unfold technique, some regular shapes with simple textures were unfolded and the deformations undergone by the textures were computed. A total of three shapes were constructed using a commercial mesh processing and editing software (Rhinoceros, McNeel North America, WA, USA). These were a hemisphere, a paraboloid and a truncated ellipsoid (Fig. 4). The shapes were chosen because of their resemblance to cardiac structures of interest such as the atrium or ventricle. All shapes were given a texture consisting of stripes to aid the interpretation of the effect of the unfold. The texture direction was either in the same direction as the shape's axis (hemisphere) or orthogonal to it (paraboloid and ellipsoid). It is not always possible to define perfectly straight lines in the texture (see truncated ellipsoid in Fig. 4) due to their intersection with the arrangement of the mesh's vertices. Each shape was unfolded by mapping its border to that of the unfolded 2D square. As all three shapes are open, borders were already present and thus a cut as shown in Fig. 3 was not necessary. Distortion in distance due to the unfold was mapped on each shape's unfolded 2D square. To compute this distortion map, for every vertex on the unfold, the ratio 2D/3D of the mean distance to adjacent vertices was plotted. This illustrates the distortion undergone by every point neighbourhood on the unfold and thus demonstrate which regions are distorted more than others.

#### 2.2.2 Glass-heart phantom

**Fig. 5:**
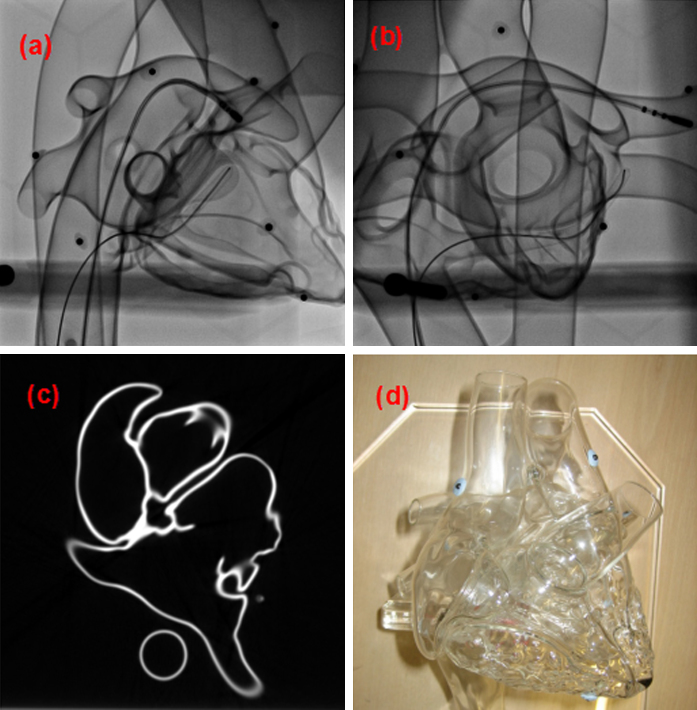
Images of the glass-heart phantom used to obtain ground truth measurements on distances. Clockwise from left: (a and b) X-ray projection images. (c) A mid-section slice through a CT scan and (d) a photographic image.

**Fig. 6:**
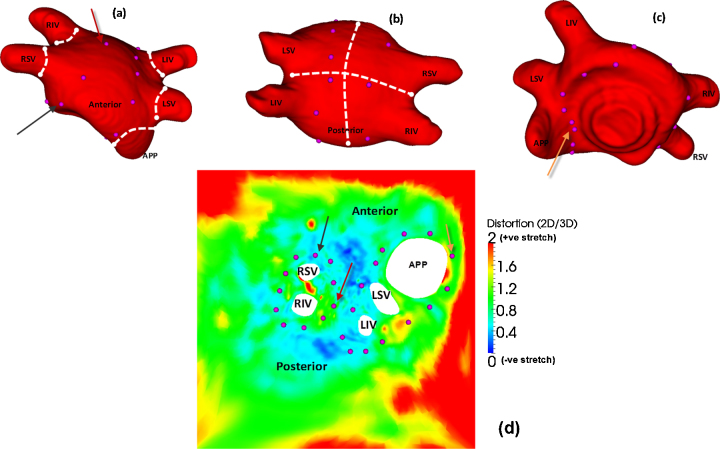
3D LA surface extracted from glass heart phantom CT scan. For ground truth testing, various measurements physically and on the unfold: (a) diameters of each vein and appendage, (b) measurements made across the roof. These are distances between the mid-anterior to mid-posterior and mid-left to mid-right, (c) basal view. (d) Distortion map of glass heart phantom illustrating distortion ratios of distances in 2D/3D. Landmarks are plotted on LA shell and unfold and correspondences are indicated using coloured arrows. (For interpretation of the references to colour in this figure legend, the reader is referred to the web version of the article.)

It is important that distances between points on the 2D unfold are comparable to actual distances in physical space and thus validated against ground truth. To validate this, a glass heart phantom (Farlow's Scientific Glassblowing, Grass Valley, CA, USA) was imaged (Fig. 5). The phantom consists of all chambers and great vessels of the heart. It was scanned by a 512 × 512 × 384 voxel CT scan with 0.68 mm × 0.68 mm ×1.00 mm voxel resolution (Brilliance iCT, Philips Healthcare, The Netherlands). A manual segmentation of the LA was obtained and a 3D surface extracted using the marching cubes algorithm. The 3D surface was cut at the mitral valve annulus (Fig. 3) and unfolded onto a 2D square by mapping the border of the cut to that of the 2D square. Thirteen separate measurements were made on the phantom by three observers. The measurements were distances between locations of anatomical interest. These measurements were carefully selected by considering their repeatability and are shown in Fig. 6. The observers measured each location three times and the mean value was calculated. These were compared to their corresponding distances on the 2D unfold. Similar to the distortion map extracted for regular shapes, a distortion map for the phantom was computed to illustrate regions undergoing distortions due to the unfolding process.

#### 2.2.3 Clinical cases

##### 2.2.3.1 Patient data

14 patients (10 males and 4 females, mean age =60 ± 8 years) with symptomatic, drug refractory paroxysmal AF undergoing PV isolation with circumferential catheter ablation procedure were included in the study. Six months following catheter intervention, a cardiac MRI scan was performed which included DE-MRI scans to quantify the extent of permanent post-ablation atrial injury. 7 of these patients subsequently underwent redo ablation procedures due to recurrence of arrhythmia. The data for the redo intervention from the electroanatomical mapping system were available, including the reconstructed 3D LA surface meshes and voltage maps.

##### 2.2.3.2 MR image acquisition

All scanning was performed on a 1.5 T Achieva scanner (Philips Healthcare, The Netherlands). A 3D whole heart image was acquired by using a 3D respiratory navigated and cardiac-gated, 3D balanced steady-state free precession (b-SSFP) acquisition in a sagittal orientation with whole-heart coverage (1.3 mm × 1.3 mm × 2.6 mm acquired, 1.3 mm × 1.3 mm × 1.3 mm reconstructed, 6 min duration). The DE-MRI scan for the visualization of delayed-enhancement was a 3D ECG-triggered, free breathing inversion recovery (IR) turbo field echo (TFE) with respiratory-navigation, cardiac-gating and with whole heart coverage (0.6 mm × 0.6 mm × 4.0 mm acquired, 0.6 mm × 0.6 mm × 2.0 mm reconstructed, 3 min duration).

##### 2.2.3.3 3D LA mesh extraction from MRI

The LA was segmented from the anatomical bSSFP scan for each of the 14 patients using an automatic approach based on a statistical shape model [26]. The model is a 3D mesh built from training data and adapted to the LA anatomical MR image. The model includes PVs which are approximately 14 mm in length and with an average diameter of 12 mm. It also includes the LA appendage and its length is approximately 15 mm from LA body. The model fitting error reported in [23] is 0.72 mm over 42 datasets. For our work, the automatic segmentation was verified by a human rater and manual corrections were made whenever necessary.

##### 2.2.3.4 Unfolding

**Fig. 7:**
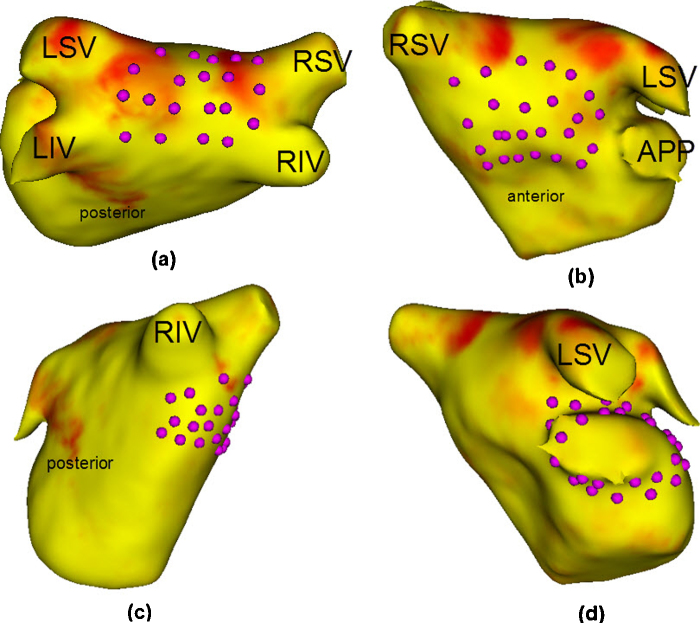
The LA surface is subdivided into segments for comparing regional distortions. Points are selected to show the indicated region. Clockwise from left: (a) roof, (b) anterior, (c) right and (d) left. The points on the posterior were selected in a similar way as on the anterior side shown above.

Each of the 14 MRI-derived LA surface meshes was unfolded using the methods described in Section 2.1. To assess the amount of distortion during the unfold process a quantitative and qualitative analysis of distortion was carried out. Quantitative analysis compared distortion in distances and areas between 3D and 2D on the MRI-derived 3D meshes. For distances, the geodesic distance between points on 3D to their mapped distance in 2D was compared. For areas, areas of regions in 3D were compared to their mapped regions in 2D. As the deformations are non-linear with every region of the atrium undergoing different amounts of deformation, the atrium was sub-divided into five distinct regions. These regions were the left and right sides, the anterior and posterior ends and the roof. Fig. 7 illustrates these regions with the points selected to perform the analyses. Distances between each pairs of points were considered and areas enclosed by every possible triangle using these points were considered. Subdividing the LA into regions allowed a segmental comparison of distortions in lengths and area. For a qualitative analysis of distortion in clinical cases, distortion maps were generated in each case. Furthermore, distortion was also visualized using texture mapping. Regular textured patterns such as a checker-board or striped patterns were generated in each LA 3D mesh and then mapped to 2D. Deformations in the textures were visualized and assessed at anterior, posterior and roof sections of LA. The 2D unfold was further investigated and evaluated to see if it could be used to select ablation lesions. Lesions were marked circumferentially around each pulmonary vein. Their mapping to 3D was validated by comparing each selected point's distance to the vein ostium in 2D and 3D. This investigated whether the unfold map overlaid with soft tissue information from DE-MRI can be accurately used to map ablation lesion points.

##### 2.2.3.5 Clinical applications

As a proof-of-concept, the clinical utility of the unfolding technique was demonstrated in three different ways. Firstly, the technique was used to display DE-MRI information (using all 14 clinical data sets); secondly, it was used to display electrical information by unfolding 3D LA meshes derived from electroanatomical mapping systems (using the 7 redo data sets); and finally, it was used to display catheter tip positions.

## 3 Results

### 3.1 Regular shapes

**Fig. 8:**
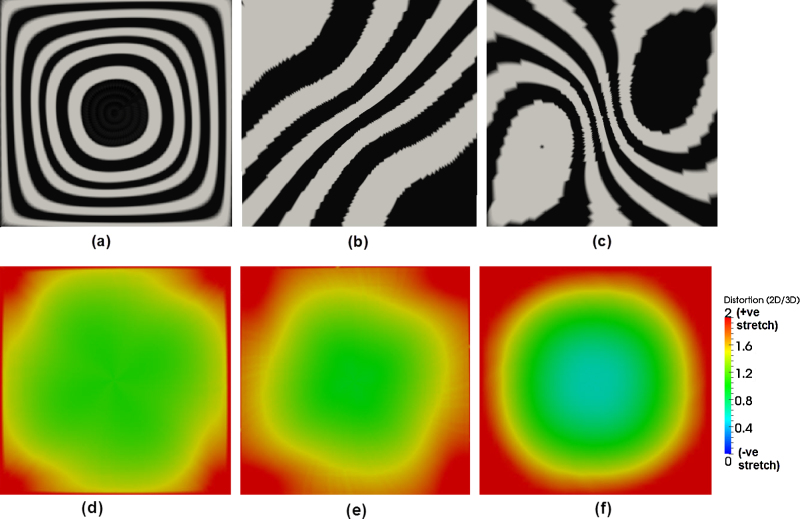
Regular geometrical shapes unfolded from (a) hemisphere, (b) paraboloid and (c) truncated ellipsoid. For these shapes refer to also Fig. 4. (d) Distortion map for hemisphere, (e) distortion map for paraboloid, (f) distortion map for truncated ellipsoid.

Mapping regular texture patterns from the 3D to 2D for regular shapes gives an understanding of the nature of deformation due to the unfold expected on a cardiac chamber such as the LA. For the open hemisphere, the circular border was mapped to a square border on the 2D unfold. This deformation of a circle to a square is evident in the deformation undergone by the texture in Fig. 8(a), with the circles stretched to fill the square's corners. Deformations appear to be close to linear throughout except at the corners of the 2D unfold. In the open paraboloid, textures were oriented orthogonally to the circular border. These texture deformations illustrate the non-linearity of the underlying transformation (see Fig. 8(b)) – the corners generally experience greater stretching. The wavy deformation in the texture exhibits this phenomenon. It is important to note why the stripes run along the diagonal of the square as opposed to being horizontal or vertically aligned. This is simply because of how the sections of the circular border in 3D match with the sections of the 2D square on unfold. Deformation in textures on the truncated ellipsoid transformation (see Fig. 8(c)) illustrate that shapes which enclose more space undergo greater deformation simply because a larger surface area now needs to be fitted within a constrained space of the 2D square. Distortion maps were also generated for each shape and given in Fig. 8(d)–(f). These maps illustrate increased stretching going from the hemisphere to ellipsoid.

### 3.2 Glass-heart phantom

**Fig. 9:**
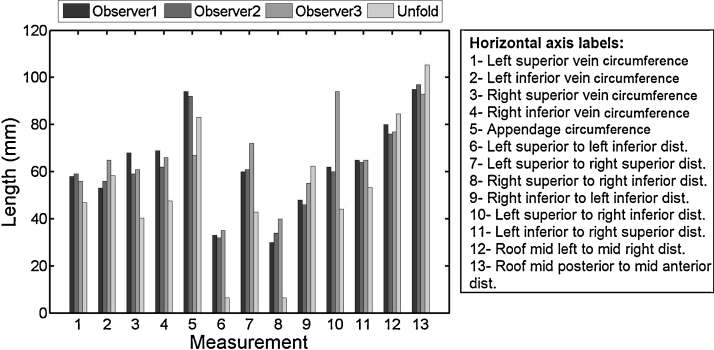
Comparison of distances of anatomical importance measured by three observers on the glass-heart phantom and the 2D unfold. For distances 12 and 13, refer to Fig. 6(b).

Physical distances on the glass-heart phantom were compared to distances measured on the 2D unfold. There are two important considerations – whether the physical measurements themselves are repeatable and whether there is intra-observer variation. It was thus important to select anatomical regions that can be distinctly located both on the phantom and unfold. The measurements obtained from observers showed that there was good agreement between them (see Fig. 9). Furthermore, measurements on the unfold were on average within 10% of the mean of all observers’ measurements. A distortion map for the phantom was computed by plotting the ratio of distance in 2D to 3D. The map is shown in Fig. 6(d). Overall, the map illustrates stretching at the borders and corners with some negative stretch or shrinking near the atrial roof. Regions near the ostium of PVs are mostly preserved with ratios close to 1.

### 3.3 Clinical cases

**Fig. 10:**
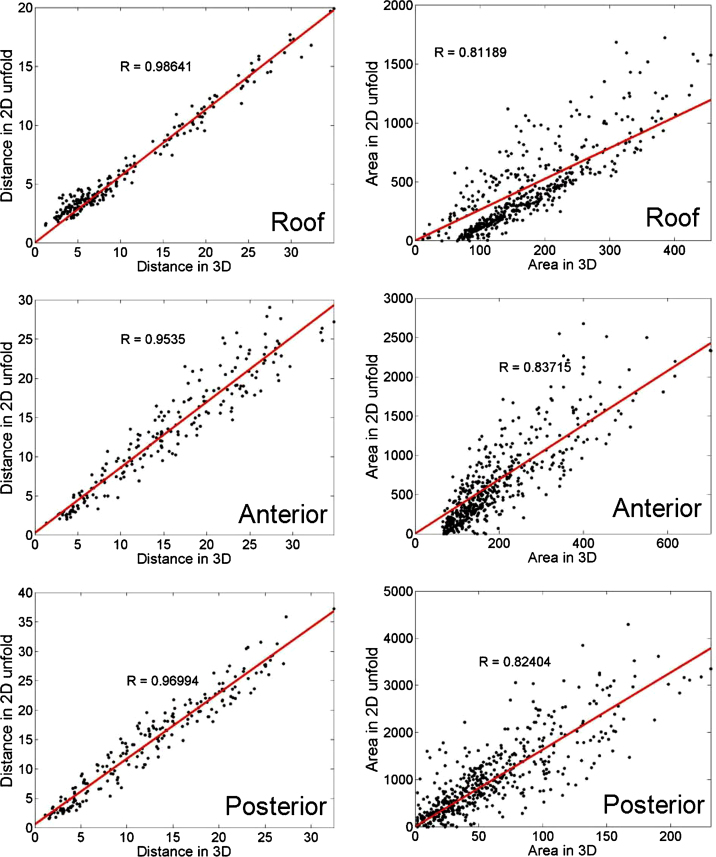

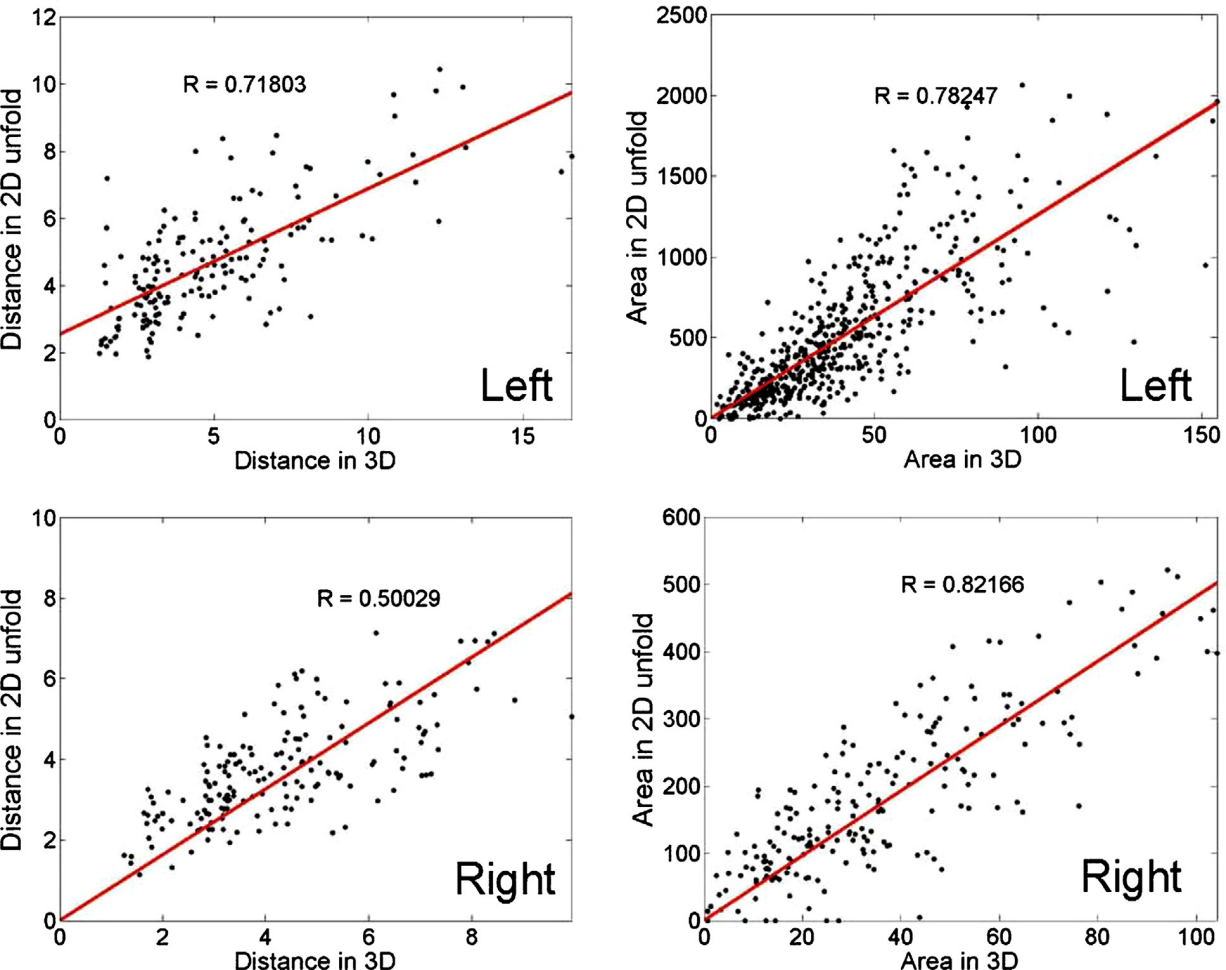
A segmental evaluation of the distortion in the geodesic distance (left column) and area (right column) due to mapping from 3D to 2D unfold. The line of best-fit is indicated together with *R*-statistic.

**Fig. 11:**
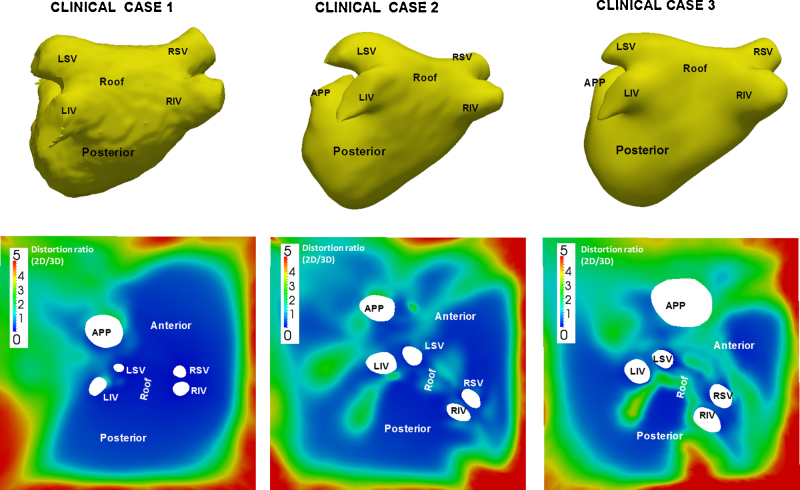
Distortion maps illustrate accuracy of the unfolding process in three clinical cases. Distortion is represented as ratio of distance in 2D to 3D and is mapped using a colour scale ranging from 0 (−ve stretch) to 5 (+ve stretch). Abbreviations for veins – by convention LSV, RSV, LIV, RIV. (For interpretation of the references to colour in this figure legend, the reader is referred to the web version of the article.)

To analyze distortions caused by the mapping process, geodesic distances on the 3D surface and their mapping on 2D are compared quantitatively in Fig. 10 (see left column) and also illustrated using distortion maps in Fig. 11. Distances are generally well preserved in the roof, posterior and anterior sections of the LA (*R* > 0.9 and low ratios in distortion maps). The right and left sides experience greater stretching because a large section of the cut is in the vicinity, whereas this was not the case with points on the roof and anterior/posterior segments. Recall that the boundary of the cut is stretched to fit the boundary of the 2D square. For the area comparison in Fig. 10 (see right column), regions of different sizes ranging from big to small were used for the analysis. Again the LA was subdivided into separate segments and each segment analyzed separately. Smaller regions across every segment show good preservation of their areas from 3D to 2D, but for larger regions, there is distortion. This is expected as small distortions within small sections of a larger region cumulate to give a resulting larger distortion.

**Fig. 12:**
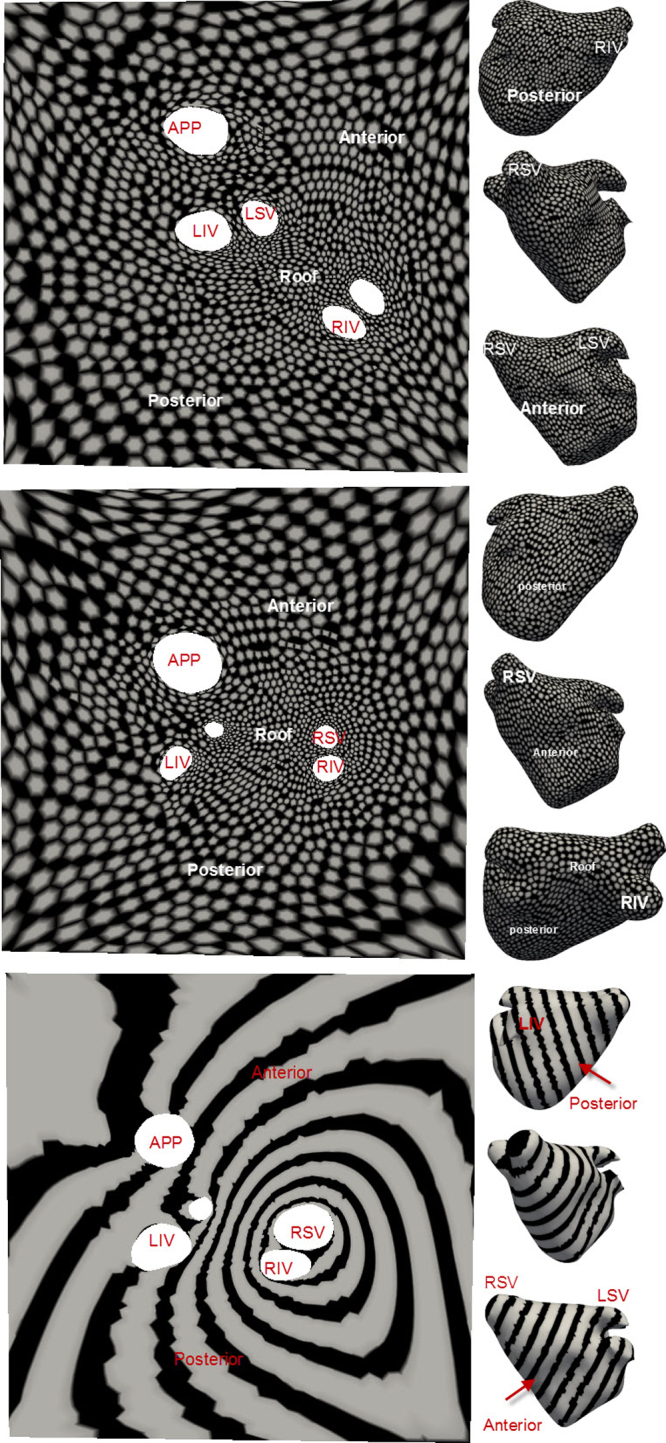
The nature of deformation due to the unfold process from 3D to 2D is analyzed here using regular texture patterns. Deformations are seen on two separate LA models with a spotted texture (top and middle row) and striped texture (bottom row). The LA in each case is shown in three different views (anterior, posterior and right side). Abbreviations for veins – by convention LSV, RSV, LIV, RIV.

The unfolding process on clinical cases is also validated using texture mapping. Textures drawn on the 3D mesh were mapped to their respective 2D unfold. As textures on the 3D are generated by utilizing the underlying mesh vertex grid, perfectly regular patterns can be difficult to achieve due to irregularity in the mesh structure, i.e. some vertices have 3, 5 or 7 neighbours depending on the 3D mesh reconstruction process. This produces small artefacts in the textures in 3D (see gaps in spotted texture in Fig. 12 and broken lines on the striped texture). Nevertheless, the nature of the deformation is demonstrated in these images and it is clear that the unfolding process of mapping a circular-like border of the cut in the annulus (see Fig. 3) to a 2D square results in larger distortions at the edge of the unfold. However, critical areas such as the roof and PV ostia are least affected and this is a desirable result in the context of LA ablation.

**Fig. 13:**
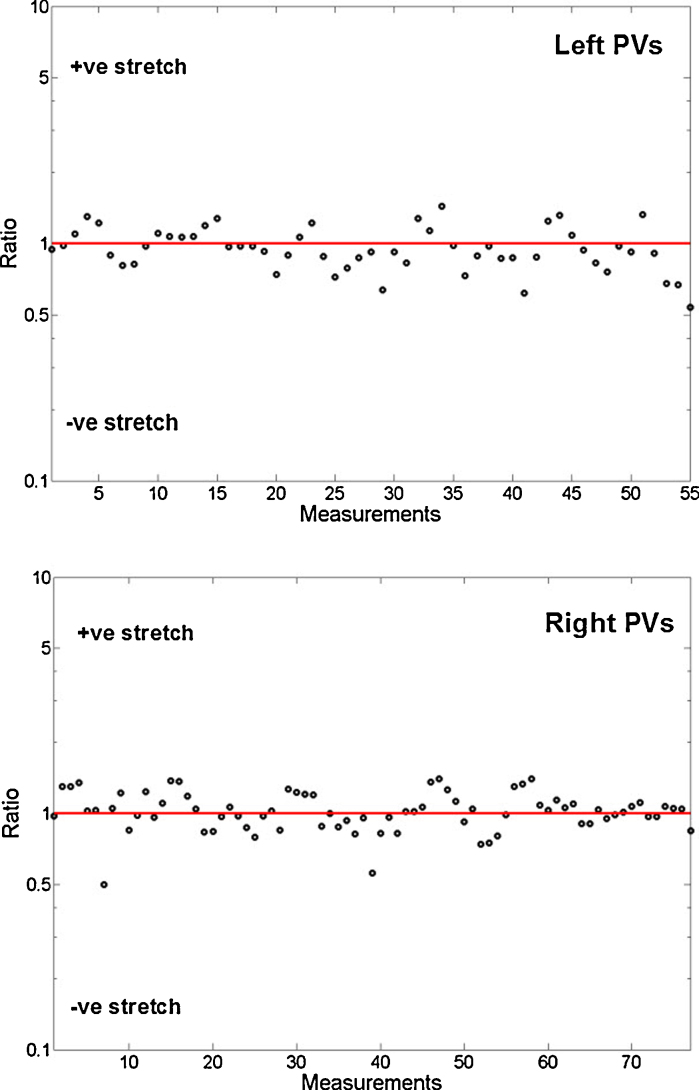
Evaluating the amount of stretch caused due to the unfold mapping to lesion points marked circumferentially on the left and right PVs. The ratio of distance in 3D to its mapped distance in 2D is plotted on a log scale. A positive (+ve) stretch means that the 3D distance is greater than in 2D and a negative (−ve) stretch otherwise.

The 2D unfold was validated as a clinical tool for marking lesion points with the added advantage of providing simultaneous soft tissue visualization. Lesions were marked circumferentially on the unfold and their distances to the vein's ostium in 2D and 3D were compared. The ratio of these distances is plotted on a log scale in Fig. 13. A positive stretch indicates that the 2D mapped distance is greater than its corresponding distance on 3D. The mean and standard deviations for left (*L*) and right (*R*) sides are: *L* = (1.032, 1.034) and *R* = (0.964, 1.039). These variations are within acceptable levels and regions around the LA ostia experience far less distortion than other areas. Thus, the 2D unfold can be used reliably for marking lesions around PVs.

## 4 Clinical applications

### 4.1 Mapping DE-MRI on the unfold for soft-tissue visualization

**Fig. 14:**
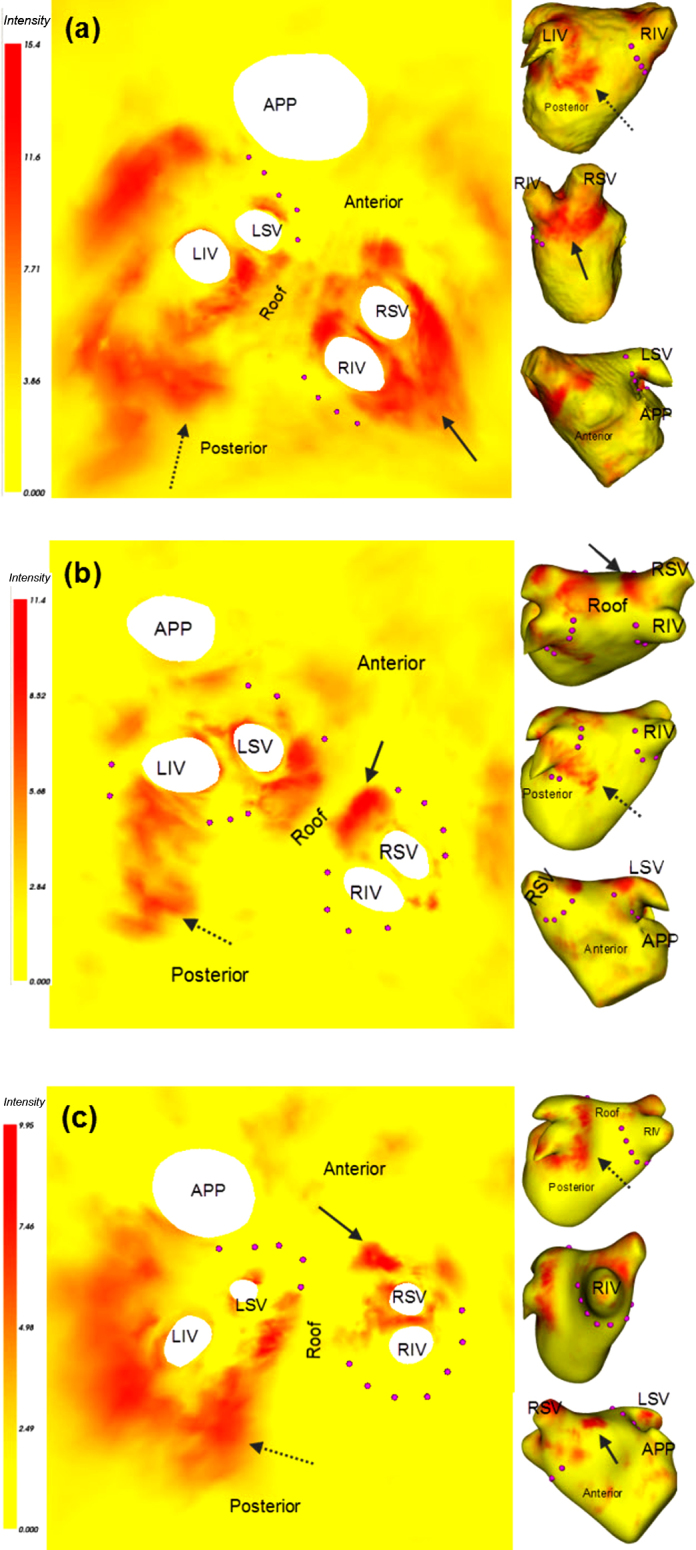
Clinical application I: mapping soft tissue information from DE-MRI to the 2D unfold in three separate clinical cases. The LA in each case is shown in three different views (posterior, side and anterior). Areas in red represent sites of previous ablation. Corresponding regions are labelled with dotted and filled arrows representing two different regions. A typical lesion set would aim to completely encircle the pulmonary veins as indicated by the stylized purple points. Abbreviations for veins – by convention LSV, RSV, LIV, RIV. (For interpretation of the references to colour in this figure legend, the reader is referred to the web version of the article.)

DE-MRI for imaging RF ablation lesions is a rapidly expanding field of research which seeks to allow a more in-depth characterization of lesions and recent studies have investigated this [27,28]. Currently, it is possible to visualize the shape and size of circumferential and linear lesion sets on the 3D LA surface. The DE-MRI image intensities due to lesions can be projected onto the 3D LA surface using the technique described in [29] which is essentially a maximum intensity projection (MIP) method. It is a two-step process. The first step involves registering the surface with the DE-MRI image allowing both the surface and DE-MRI image to be in the same co-ordinate system. Once fused, DE-MRI image intensity is mapped to the 3D LA surface using MIP. Once on the 3D surface, DE-MRI image intensity can be mapped to the 2D unfold. Three examples from clinical cases are shown in Fig. 14. DE-MRI image intensity in Fig. 14 is mapped on a yellow to red linear colour scale with red indicating scar. Circumferential scarring around the PVs is expected. Gaps in scarring can lead to PV reconnection and recurrence of arrhythmia. The entire distribution of the scarring is revealed in the unfold, as shown in Fig. 14, and gaps are easily evident. The electrophysiologist will seek to close these gaps by re-ablating these areas. In Fig. 14 points have been hypothetically selected to close these gaps (see purple points).

### 4.2 Endocardial voltage mapping

**Fig. 15:**
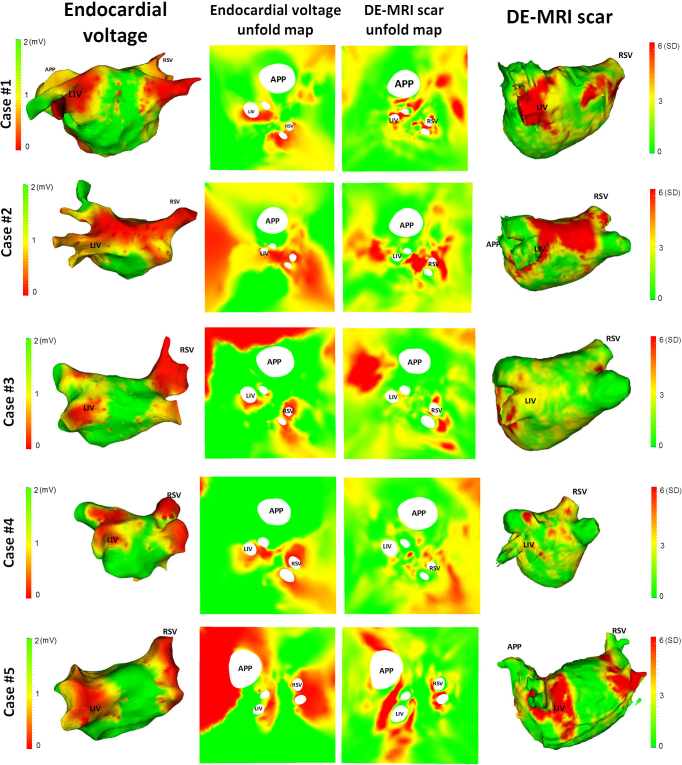
Clinical application II: unfold on 3D electroanatomical LA mesh obtained from mapping systems. Five clinical re-do ablation cases are shown here with electroanatomical map (column 1) and its unfold (column 2) and compared with each case's pre-ablation MRI reconstructed 3D mesh with DE-MRI scar enhancement (column 4) and its unfold (column 3). Note low voltage in milli-volts (mV) is comparable to high intensity in DE-MRI represented as standard deviations (SD) from healthy tissue intensity. Abbreviations for veins – by convention LIV, RSV, APP–appendage. (For interpretation of the references to colour in this figure legend, the reader is referred to the web version of the article.)

The majority of cardiac electrophysiologists utilize an electroanatomical mapping system to guide the ablation procedure. The mapping system, such as CARTO, is able to acquire unipolar or bipolar readings of voltages on the endocardium using the catheters present inside the heart. Prior to voltage mapping, the 3D LA geometry of the patient is collected using a tracked catheter. The geometry is reconstructed from a point cloud recorded by the catheter's tip. Endocardial voltage is mapped, displayed and visualized on the 3D LA geometry. In a recent study [28] it was shown that voltage and scar have an important relationship and thus it is useful to correlate and visualize the two especially for re-do procedures. However, there is an important complexity when correlating voltage and scar. The 3D electroanatomical geometry is far less detailed than the 3D LA scar mesh obtained from MRI, owing to high resolution in MRI images. Using the 2D unfold, the complexity of the two dissimilar geometries is reduced and correspondences can be made. In Fig. 15, several re-do clinical cases are illustrated. In each case, the endocardial voltage maps are unfolded and compared with pre-ablation DE-MRI scar imaging. Note low voltage is comparable to high intensity DE-MRI and thus the colour scale in voltage maps are inverted (red to green). The 2D unfold reduces the complex difference between the two geometries and presents similar looking maps in each case whereby voltage and scar can be compared. Lesion formation circumferentially on each side is compared with low voltage areas. Cases 1–4 demonstrate low voltage areas on each side and correspond well with significant DE-MRI enhancement in those areas.

### 4.3 Catheter visualization and navigation

**Fig. 16:**
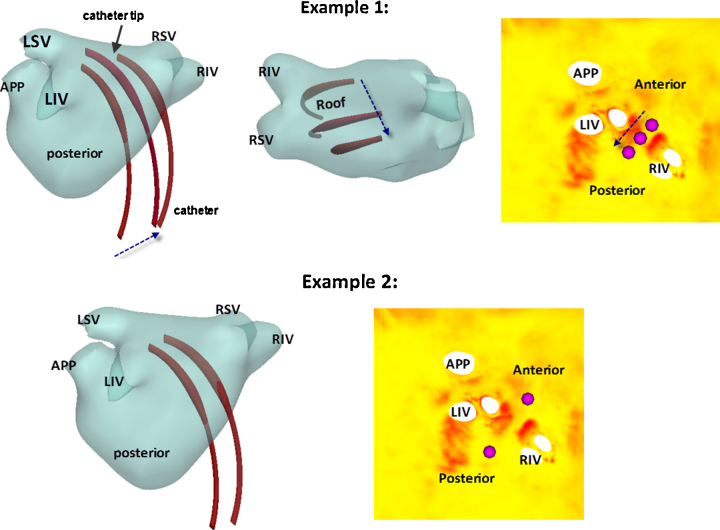
Clinical application III: unfold in catheter visualization and navigation: example 1 shows an instance where the catheter is manoeuvred from anterior to posterior along the roof (direction indicated by dashed arrow). In the 3D posterior LA view, it is unclear whether the catheter is nearer to posterior or anterior wall until the 3D model is re-oriented (roof view). The unfold shows the catheter tip as it moves along the roof (pink stylized points) and there is no ambiguity about its location. Example 2 shows a similar situation where it is unclear whether the catheter is on the posterior or anterior wall in the 3D view and the electrophysiologist is able to confirm its location using the 2D unfold view. (For interpretation of the references to colour in this figure legend, the reader is referred to the web version of the article.)

During ablation procedures, electroanatomical mapping systems display live catheter positions on a 3D LA mesh. In most instances, it is unclear to an electrophysiologist whether the catheter tip is on the posterior or anterior side of the wall unless the 3D mesh is interacted with and viewed from different angles. The unfold can display catheter tip position in a variety of ways depending on whether the catheter is near or in contact with the wall. Without any ambiguity or interaction, using the unfold the electrophysiologist is able to confirm which side of the atrium the catheter is located at. In Fig. 16, two examples are illustrated to demonstrate that the unfold can be used simultaneously with the 3D mesh to provide useful positional information of a catheter during an ablation procedure.

## 5 Discussion

The method of LA 3D surface parameterization implemented in this work computes optimal locations for 3D mesh vertices in a pre-defined 2D parameter space. The optimality condition is achieved by minimizing a linear combination of functions that preserve angles and areas of each mesh patch subject to boundary conditions. The primary aim was to use the method to unfold the left atrium of the heart to support the management of patients with atrial fibrillation. The method was validated using synthetic data and physical phantom data. It was then applied to patient data derived from MRI and electroanatomical mapping systems to demonstrate clinical utility.

The mapping procedure implemented in this work provides a one-to-one mapping. Points selected on the unfold can be located on the 3D and vice versa as demonstrated by the stylized points in Fig. 14. Generation of the unfold 2D map from the 3D shell is fast, requiring less than 3 min on a 2.5 GHz PC. Once the unfold is generated, using internal mapping lookup tables for locating and mapping points between 2D and 3D is virtually instantaneous. Cutting the LA shell at the annulus is also quick and requires less than 5 min.

Distortions due to the unfold were found to be unavoidable and this is simply due to the restrictions imposed on the unfolding process through boundary conditions (i.e. 2D square). By performing segmental comparisons of distance and area, it was found that regions closer to the location of the cut in the mitral valve annulus and on the left and right sides of the veins experienced the greatest distortion and stretching. However, the amount of stretching involved does not distort the lesion pattern to an extent that it is unsuitable for visualization. This is clearly demonstrated in the lesion maps of Fig. 14 where lesion formations around the veins are preserved. The most critical areas lie in and around PVs and roof region and this work demonstrated that distortion in these regions was not high.

To understand how distortions of the nature seen in the LA unfold might affect an end-user, it is important to consider a scenario where the user uses the unfold to select lesions. In such cases, endocardial voltage or DE-MRI enhancement information on the unfold is used to decide lesion positions. Points selected on the unfold are made relative to features visible in the unfold itself such as distorted lesion or voltage patterns. As these features are themselves distorted to a certain extent, the end-user would have little difficulty identifying locations of interest once the nature of distortion was understood and appreciated.

Several proof-of-concept clinical applications were demonstrated. Visualizing scar tissue from DE-MRI on the 2D unfold reveal gaps in ablation lesions and provides useful information for re-do procedures. Unfolding of electroanatomical maps was also presented, in particular for displaying endocardial voltage information. Finally, the 2D unfold was demonstrated as a useful utility during catheter-tip visualization on 3D transparent models. Ambiguous positions in 3D require interaction with the model – an example of points lying on the posterior and anterior wall is demonstrated. On the 2D unfold, such ambiguity is removed as the posterior and anterior walls can be simultaneously visualized.

### 5.1 Limitations

The proposed method has a number of limitations. The cut made at the mitral valve annulus of the 3D LA mesh during method initialization imposes a limitation on the unfold. Since it is desirable for the PV ostia to lie centrally and approximately symmetrically on the 2D unfold, the cut on the left and right sides should be such that its geodesic distance from left and right PVs are alike. The same applies to the posterior and anterior sides and their distance from LA roof. However, obtaining the optimal cut manually can be difficult given the above constraints, and further, the folding of the LA surface at the appendage makes it challenging. Consequently, in some instances, a few sub-optimal cut configurations were explored before generating the 2D unfold. Operator bias and thus inter-observer variation was not completely eliminated.

There were limitations in the methods used for validating the 2D unfold. A 2D unfold of the glass-heart phantom was generated and distances on the phantom were compared, i.e. physical versus 2D. Point correspondence between physical and virtual 3D/2D was established in this work using simple visual assessment, i.e. for a point selected on the glass-heart, its position on the 3D LA mesh was manually selected. There are novel ways of determining where exactly a point lies in physical space and this is by employing magnetic trackers or some form of imaging. Establishing correspondence manually introduces errors but this was minimized by selecting points in regions that were anatomically distinct.

The 2D unfold was validated in this work by quantifying distortion. Distortion maps were generated for various shapes and LA models. In these maps, the mean distance to adjacent neighbours in a mesh is considered. Points farther away from immediately adjacent neighbours are not considered. Given that distortion tends to be greater when computed over larger distances and areas, this can be a limitation of these maps. However, distortion over larger regions was investigated in the segmental evaluation of clinical cases with plots in Fig. 10. Data points have a wider spread and deviate from the linear relationship for larger distances and areas indicating greater distortion.

All 2D unfold explored in this work was on a square. The square was chosen as it can be more convenient to work with on a flat visual display unit. Other shapes are not explored and one good candidate for mapping would be on a circle. Mapping on the square imposes significant distortions at corners; a circle offers smoother gradients at its edges and is thus expected to have lesser distortions. However, representing it on a flat visual display unit can present some difficulties especially when zooming in whereby a section of circle can get clipped.

The 2D unfold implemented in this work was validated using various techniques. It has potential uses in many areas including LA ablation procedures for simplifying visualization. However, it was not validated within a real-time ablation procedure. This would involve unfolding either the electroanatomical maps acquired during a procedure or unfolding the pre-ablation MRI data. The 2D unfold map would be assessed to see if it offered any advantages over a 3D map.

## 6 Conclusions

Procedural guidance using a 3D mesh can be cumbersome and demands constant interaction with the geometry. The use of planar maps with point-by-point catheter technologies (manual or robotic) could decrease the complexity of navigation and even improve navigation by less experienced operators. The proposed work investigates an approach to flatten and visualize 3D LA geometry along with scar and/or endocardial voltage information on a 2D planar map. The 2D unfold is both quantitatively and qualitatively validated and several clinical proof-of-concept clinical applications are demonstrated. Future work will look into employing planar maps in live catheter procedures and to evaluate clinical utility.

## References

[1] Floater M.S., Hormann K. (2005). Surface parameterization: a tutorial and survey. Advances in Multiresolution for Geometric Modelling.

[2] Floater M.S. (1997). Parametrization and smooth approximation of surface triangulations. Computer Aided Geometric Design.

[3] Floater M.S. (2003). Mean value coordinates. Computer Aided Geometric Design.

[4] Floater M.S., Reimers M. (2001). Meshless parameterization and surface reconstruction. Computer Aided Geometric Design.

[5] Tutte W.T. (1963). How to draw a graph. Proceedings of the London Mathematical Society.

[6] Sheffer A., de Sturler E. (2001). Parameterization of faceted surfaces for meshing using angle-based flattening. Engineering with Computers.

[7] Lévy B., Petitjean S., Ray N., Maillot J. (2002). Least squares conformal maps for automatic texture atlas generation. ACM Transactions on Graphics (TOG).

[8] Desbrun M., Meyer M., Alliez P. (2002). Intrinsic parameterizations of surface meshes. Computer Graphics Forum.

[9] Hurdal M.K., Stephenson K., Bowers P., Sumners D.W., Rottenberg D.A. (2000). Coordinate systems for conformal cerebellar flat maps. NeuroImage.

[10] Haker S., Angenent S., Tannenbaum A., Kikinis R., Sapiro G., Halle M. (2000). Conformal surface parameterization for texture mapping. IEEE Transactions on Visualization and Computer Graphics.

[11] Gu X., Wang Y., Chan T.F., Thompson P.M., Yau S.T. (2004). Genus zero surface conformal mapping and its application to brain surface mapping. IEEE Transactions on Medical Imaging.

[12] Wang Y., Shi J., Yin X., Gu X., Chan T.F., Yau S.T. (2012). Brain surface conformal parameterization with the ricci flow. IEEE Transactions on Medical Imaging.

[13] Joshi A.A., Shattuck D.W., Thompson P.M., Leahy R.M. (2007). Surface-constrained volumetric brain registration using harmonic mappings. IEEE Transactions on Medical Imaging.

[14] Dale A.M., Fischl B., Sereno M.I. (1999). Cortical surface-based analysis. I. Segmentation and surface reconstruction. NeuroImage.

[15] Cerqueira M.D., Weissman N.J., Dilsizian V., Jacobs A.K., Kaul S., Laskey W.K. (2002). Standardized myocardial segmentation and nomenclature for tomographic imaging of the heart a statement for healthcare professionals from the cardiac imaging committee of the Council on Clinical Cardiology of the American Heart Association. Circulation.

[16] Go A.S., Hylek E.M., Phillips K.A., Chang Y., Henault L.E., Selby J.V. (2001). Prevalence of diagnosed atrial fibrillation in adults. Journal of the American Medical Association.

[17] Haïssaguerre M., Jaïs P., Shah D.C., Takahashi A., Hocini M., Quiniou G. (1998). Spontaneous initiation of atrial fibrillation by ectopic beats originating in the pulmonary veins. New England Journal of Medicine.

[18] Pappone C., Rosanio S., Oreto G., Tocchi M., Gugliotta F., Vicedomini G. (2000). Circumferential radiofrequency ablation of pulmonary vein ostia: a new anatomic approach for curing atrial fibrillation. Circulation.

[19] Oral H., Pappone C., Chugh A., Good E., Bogun F., Pelosi F. (2006). Circumferential pulmonary-vein ablation for chronic atrial fibrillation. New England Journal of Medicine.

[20] Karch M.R., Zrenner B., Deisenhofer I., Schreieck J., Ndrepepa G., Dong J. (2005). Freedom from atrial tachyarrhythmias after catheter ablation of atrial fibrillation: a randomized comparison between 2 current ablation strategies. Circulation.

[21] Calkins H., Brugada J., Packer D.L., Cappato R., Chen S.A., Crijns H.J. (2012). 2012 HRS/EHRA/ECAS expert consensus statement on catheter and surgical ablation of atrial fibrillation. Europace.

[22] Ma Y., Karim R., Housden R.J., Gijsbers G., Bullens R., Aldo C. (2012). Cardiac unfold: a novel technique for image-guided cardiac catheterization procedures. Information Processing in Computer-Assisted Interventions.

[23] Pinkall U., Polthier K. (1993). Computing discrete minimal surfaces and their conjugates. Experimental Mathematics.

[24] Press W.H., Teukolsky S.A., Vetterling W.T., Flannery B.P. (1986). Numerical recipes.

[25] Fabri A., Pion S. (2009). CGAL: the computational geometry algorithms library. Proceedings of the 17th ACM SIGSPATIAL international conference on advances in geographic information systems.

[26] Ecabert O., Peters J., Schramm H., Lorenz C., von Berg J., Walker M.J. (2007). Automatic whole heart segmentation in static magnetic resonance image volumes. Medical image computing and computer-assisted intervention – MICCAI 2007.

[27] Arujuna A., Karim R., Caulfield D., Knowles B., Rhode K., Schaeffter T. (2012). Acute pulmonary vein isolation is achieved by a combination of reversible and irreversible atrial injury after catheter ablationclinical perspective evidence from magnetic resonance imaging. Circulation: Arrhythmia and Electrophysiology.

[28] Malcolme-Lawes L., Juli C., Karim R. (2013). Automated analysis of atrial late gadolinium enhancement imaging that correlates with endocardial voltage and clinical outcomes: a 2-center study. Heart Rhythm.

[29] Knowles B.R., Caulfield D., Cooklin M., Rinaldi C.A., Gill J., Bostock J. (2010). 3-D visualization of acute RF ablation lesions using MRI for the simultaneous determination of the patterns of necrosis and edema. IEEE Transactions on Biomedical Engineering.

